# Modified Palatal Roll Flap: A Novel Technique for Horizontal Ridge Augmentation

**DOI:** 10.7759/cureus.58890

**Published:** 2024-04-24

**Authors:** Kanishk Srivastava, Sunil Kothawar

**Affiliations:** 1 Dentistry, Manipal Academy of Higher Education, Manipal, IND; 2 Oral Implantology, Asian Institute of Advanced Dentistry-AIAD, Hyderabad, IND; 3 Oral and Maxillofacial Surgery, Dr. M.G.R. Educational And Research Institute, Chennai, IND; 4 Oral Pathology and Oral Implantology, Asian Institute of Advanced Dentistry-AIAD, Hyderabad, IND

**Keywords:** aesthetic zone, palatal roll flap, alveolar ridge augmentation, dental implantology, minor oral surgery

## Abstract

The Abrams’ palatal roll technique has undergone significant changes over time and is routinely utilized to widen peri-implant soft tissues in the maxillary aesthetic zone. The described technique involves the use of a de-epithelized pedicled connective tissue flap from the palate and rolling it under the labial oral mucosa to increase the soft tissue bulk. It is an easy and efficient technique that improves gingival thickness and contour in the aesthetic region and is a great substitute for areas lacking gingival soft tissue. Furthermore, this technique eliminates the need for a second surgical site to harvest the connective tissue graft for soft tissue augmentation. This study reports a clinical case where the modified roll technique was used for horizontal ridge augmentation.

## Introduction

In the current era, aesthetics have become the most important aspect of dental treatment, as patients demand a more natural and unidentifiable dental treatment, especially for the replacement of missing teeth. Dental implants with soft tissue augmentation are considered the best possible rehabilitation for lost teeth [1]. After tooth loss, there is a collapse of the alveolar ridge, limiting the availability of bone for implant placement [2], which can result in up to 60% reduction of the ridge within 2 years of tooth loss [3]. Bone loss also results in deficiencies in soft tissue contours, which must be corrected with soft tissue augmentation [4-7]. This palatal roll technique is not new and has been well-documented in the literature [8-10]. The modified palatal roll flap technique covers a greater amount of rollable connective tissue, thereby minimizing bone exposure and preserving the epithelial contour over the implant, also allowing for easier postoperative recovery.

## Case presentation

A 19-year-old male patient was referred to an area charitable dental hospital for implant-based rehabilitation of the upper right central incisor, which had fractured due to trauma. Medical examination revealed no alterations, whereas intraoral examination revealed a fractured upper-right-central incisor. Consequently, implant placement was performed using the socket shield technique and grafting of the jumping distance was performed. After 5 months, the patient was recalled for the prosthetic phase of the treatment. Upon intraoral examination, it was noticed that there was a labial soft tissue depression over the implant region, i.e., Seibert Class I defect of the ridge. Therefore, in this case, the palatal roll flap technique was used.

**Figure 1:**
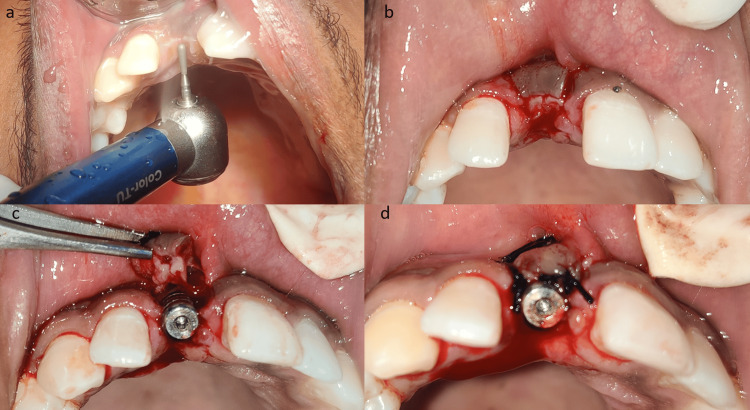
Intraoral images showing the surgical technique for palatal roll flap a) De-epithelization of palatal mucosa, b) Incision for palatal roll flap, c) Flap rotated such that de-epithelized mucosa meets connective tissue, d) Surgical site sutured with 3-0 black silk.

Adequate local anesthesia was achieved with 2% lignocaine with 1: 80,000 dilution of adrenaline. The implant cover screw was palpated by using a dental probe. With slight palatal extension, the epithelium overlying the cover screw was de-epithelized with the help of an air rotor, running it superficially over the mucosa with a pear-shaped diamond abrasive bur (Figure 1a) until pinpoint capillary bleeding was evident. Using a number 15 blade at the palatal end of the de-epithelized mucosa, a horizontal incision was made, and then two vertical releasing incisions were placed on either side of the implant, sparing the marginal gingiva (Figure 1b). A full-thickness flap was elevated and rolled upon itself, such that the de-epithelized mucosa met the connective tissue underneath the flap (Figure 1c). A horizontal matrix suture was placed to hold the rolled palatal flap in the desired position using a 3-0 black-silk suture. A healing abutment was placed over the implant and the surgical site was closed with simple interrupted sutures using 3-0 black silk (Figure 1d).

**Figure 2:**
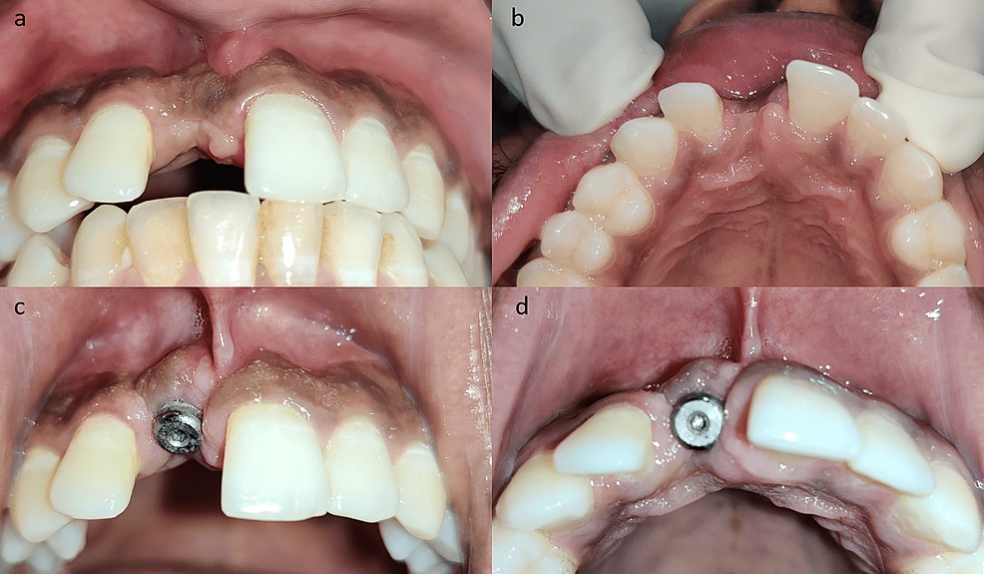
Intraoral images showing increased labial soft tissue thickness at the implant site a, b) Preoperative images showing soft tissue defect at the implant site. c, d) Postoperative images showing horizontal ridge augmentation with palatal roll flap.

The patient was prescribed routine antibiotics and painkillers postoperatively for 3 days and recalled after 10 days for suture removal. After another 5 days, the patient was recalled for an open tray impression during the prosthetic phase, and intraoral examination revealed a drastically improved labial gingival soft tissue profile over the implant region (Figure 2).

## Discussion

Tooth loss results in significant atrophy of the alveolar process, with loss of hard and soft tissues [4,5]. In the process of implant rehabilitation, especially in the aesthetic zone, the loss of soft tissue contour will result in an artificial appearance of the implant prosthesis, if not addressed correctly. Abrams was the first to describe a flap technique for deformed residual edentulous [8]. Later, in 1992, Scharf and Tarnow modified Abram's roll technique and described it as a "trap-door" approach. The flap was used to reflect and preserve the epithelium that overlies the connective tissue pedicle, and the donor site was then covered with the epithelial pedicle [9]. Since then, multiple techniques have been proposed for soft tissue augmentation around implants, including subgingival connective tissue grafting, roll technique, pouch roll technique, modified roll technique, and mixed grafts [1,2,7,10]. The increase in soft tissue bulk around the implant not only improves aesthetics but also provides functional protection to the implant, thereby increasing its prognosis [1,2,10]. The mentioned modified roll flap technique uses an air rotor to de-epithelize the mucosa, and as it is a superficial abrasion over the mucosa, there is no risk of air embolism.

## Conclusions

The technique provides a vascularized graft with increased stabilization over the grafted area and definitive increases in the buccal soft tissue width. In addition to its simplicity, this technique is also predictable, reliable, less invasive, easy to perform, with minimal trauma, and provides good aesthetic outcomes.
